# Long Biliopancreatic Limb (BPL) RYGB Versus Short BPL RYGB Post-Suboptimal Initial Clinical Response of SG or Recurrent Weight Gain: A Randomized Controlled Study

**DOI:** 10.1007/s11695-025-08033-x

**Published:** 2025-07-09

**Authors:** Mohamed Abdalla Salman, Ahmed Saad, Ahmed Fahmy Omar, Mohannad Aly Fayed, Ahmed Saeed Hasan Saqr, Ahmed Abdalla

**Affiliations:** Kasralainy School of Medicine, Cairo University, Giza, Egypt

**Keywords:** Sleeve gastrectomy (SG), Suboptimal initial clinical response (SoCR), Recurrent weight gain (RWG), Conversion surgery, Roux-en-Y gastric bypass (RYGB), Long biliopancreatic limb (BPL), Short BPL

## Abstract

**Background:**

Roux-en-Y gastric bypass (RYGB) has long been one of the main metabolic and bariatric choices. It has been the most frequently used conversional procedure for a sleeve gastrectomy (SG) with suboptimal initial clinical response (SoCR) or recurrent weight gain (RWG). RYGB provides its effect through both dietary restriction and malabsorption, with efficient weight and metabolic control relating to the excluded gut. This study was designed to investigate the effect of long biliopancreatic limb (BPL) RYGB versus short BPL RYGB post-SoCR or RWG of SG on weight loss and metabolic profile.

**Patients and Methods:**

This is a randomized controlled trial that included patients who had undergone SG with SoCR or RWG and were equally enrolled in the long BPL (LRYGB-LBPL) group and the short BPL group (LRYGB-SBPL). The patients were followed for 1 year, and weight loss, metabolic profile, and postoperative complications were analyzed.

The LRYGB-LBPL group showed statistically significant improvements in EBMIL%, HbA1c reduction, and HDL levels, while other outcomes showed no significant differences.

This study’s findings suggest that extending the BPL length in conversional RYGB may enhance the procedure’s effectiveness in certain aspects of weight reduction and metabolic profile, although many outcomes were similar between groups.

## Introduction

Obesity has become a global pandemic, with approximately one-third of the adult population obese or overweight. This prevalence is continuously increasing, with parallel increases in the associated medical complications such as dyslipidemia, cardiovascular disorders, and type 2 diabetes mellitus [1–3].

Metabolic and bariatric surgery (MBS) is considered the most effective intervention for achieving significant and sustained weight loss in patients with obesity, surpassing the outcomes of non-surgical treatments [4]. The most performed MBS since 2014 has been sleeve gastrectomy (SG), as announced by the International Federation for the Surgery of Obesity (IFSO), with described feasibility and satisfactory outcomes that led to continuously rising popularity [5]. However, it poses the risk of SoCR due to improper weight control or being complicated with the worsening or de novo occurrence of gastroesophageal reflux disease (GERD) [6, 7].

Roux-en-Y gastric bypass (RYGB) has long been one of the main metabolic and bariatric choices. It has been the most frequently performed conversional procedure for an SG with SoCR or RWG, and its achieved benefits are outweighing the potential risks [8, 9]. RYGB, in addition to the formation of a gastric sleeve, entails the creation of three limbs: the biliopancreatic limb (BPL), the alimentary limb (AL), and the common limb (CL), providing its effect through both dietary restriction and malabsorption, with efficient weight and metabolic control relating to the excluded gut [10].

Several studies addressed the manipulation of limb lengths and its potential impact on surgery outcomes. However, no study, as far as we know, investigated the impact of limb length variation on the conversional RYGB’s outcomes in a prospective study [11]. This study was designed to investigate the effect of long BPL RYGB versus short BPL RYGB post-SoCR or RWG of SG on weight loss and metabolic profile.

## Patients and Methods

This is a randomized controlled trial (RCT) that involved patients who had previously SG with SoCR or RWG and were recruited to our institution for conversional surgery. The study started after obtaining ethical approval from the research ethics committee. Helsinki Declaration guidelines were adhered to throughout the study.

For the calculation of the sample size, the G*Power 3.1.9.7 software was used, and calculations were done based on the comparison of the weight loss outcome between long and short BPLs RYGB found in the study by Zerrweck et al. [12]. Utilizing a two-tailed t-test for mean differences, we set the effect size (*d*) at 0.533 and an alpha level (*α*) of 0.05. The desired power was set at 0.80. The noncentrality parameter (*δ*) was calculated as 2.53, with a critical *t* value of 1.66. This resulted in the required sample size of at least 45 participants per group, totaling 90 participants. We further increased. To account for potential dropouts or unforeseen variations, we added 10 patients to each group, increasing the total sample size to 110 participants.

Patients who had had SG for treatment of obesity and its associated complications that showed SoCR, as defined by achieving a less than 50% percentage of excess weight loss (EWL%) over 18–24 months, having a body mass index (BMI) above 35 kg/m^2^ [13], RWG, as defined by weight gain that occurs after achievement of an initial successful weight loss (defined as EWL>50%) [14], or being complicated by interactable GERD, were eligible for the study. These patients were identified retrospectively from hospital records, ensuring that their weight loss outcomes were evaluated within this specific postoperative timeframe. Patients who did not agree to participate in the study were excluded. Written informed consent was obtained from each patient before being involved in the study.

The included patients were subjected to a complete clinical assessment by a multidisciplinary team. Ensuring fitness for MBS was guided by the 1991 NIH consensus [15], the American Society for Metabolic and Bariatric Surgery (ASMBS), and IFSO [16].

A simple questionnaire published in 2011 was used to assess the GERD symptoms [17]. This was used both preoperatively and at the 12-month postoperative follow-up. GERD remission was defined as a total symptom score of 0, indicating the absence of heartburn, regurgitation, and vomiting. GERD improvement was defined as a reduction in the total symptom score by at least 50% compared to baseline.

The eligible patients were equally and randomly enrolled into two groups: the LRYGB-LBPL group (with a long BPL) and the LRYGB-SBPL group (with a short BPL). Randomization was done using the closed opaque envelope method that was done by an independent hospital colleague.

After the routine patients’ preparation, RYGB surgery was performed laparoscopically under general anesthesia while patients were in the reverse Trendelenburg position. Abdominal incisions were done in the diamond-shaped five-trocar pattern, and sufficient pneumoperitoneum was achieved. The surgery was conducted as established, with a BPL length of 150 cm and an AL length of 75 cm in the patients of the LRYGB-LBPL group and respective lengths of 50–75 cm and 150 cm in the patients of the LRYGB-SBPL group.

After surgery, patients were motivated for early mobilization. They received routine postoperative care, and the required diet and supplementation regimens were provided on hospital discharge.

The patients were followed until 1 year after surgery at regular, scheduled intervals. The EWL% and the percentage of excess BMI loss (EBMIL%) were calculated as standardized [18], and comorbidity remission was judged per the ASMBS standardized outcome reporting [19].

The primary outcomes of this study were the comparative weight loss and metabolic profile in the two groups at the 1-year follow-up. The secondary outcomes were the surgery-related complications and the length of hospital stay (LOS).

## Statistical Analysis

The SPSS statistical software (IBM Corp., Armonk, NY, USA), version 28, was used to conduct the statistical analyses of the current study. Numerical data were compared using an independent or paired t-test accordingly. The chi-square test, z-test for proportion, or Fisher’s exact test were used for the comparison of the categorical values, as required. Statistical significance was indicated when the *p*-value was less than 0.05.

## Results

**Fig. 1:**
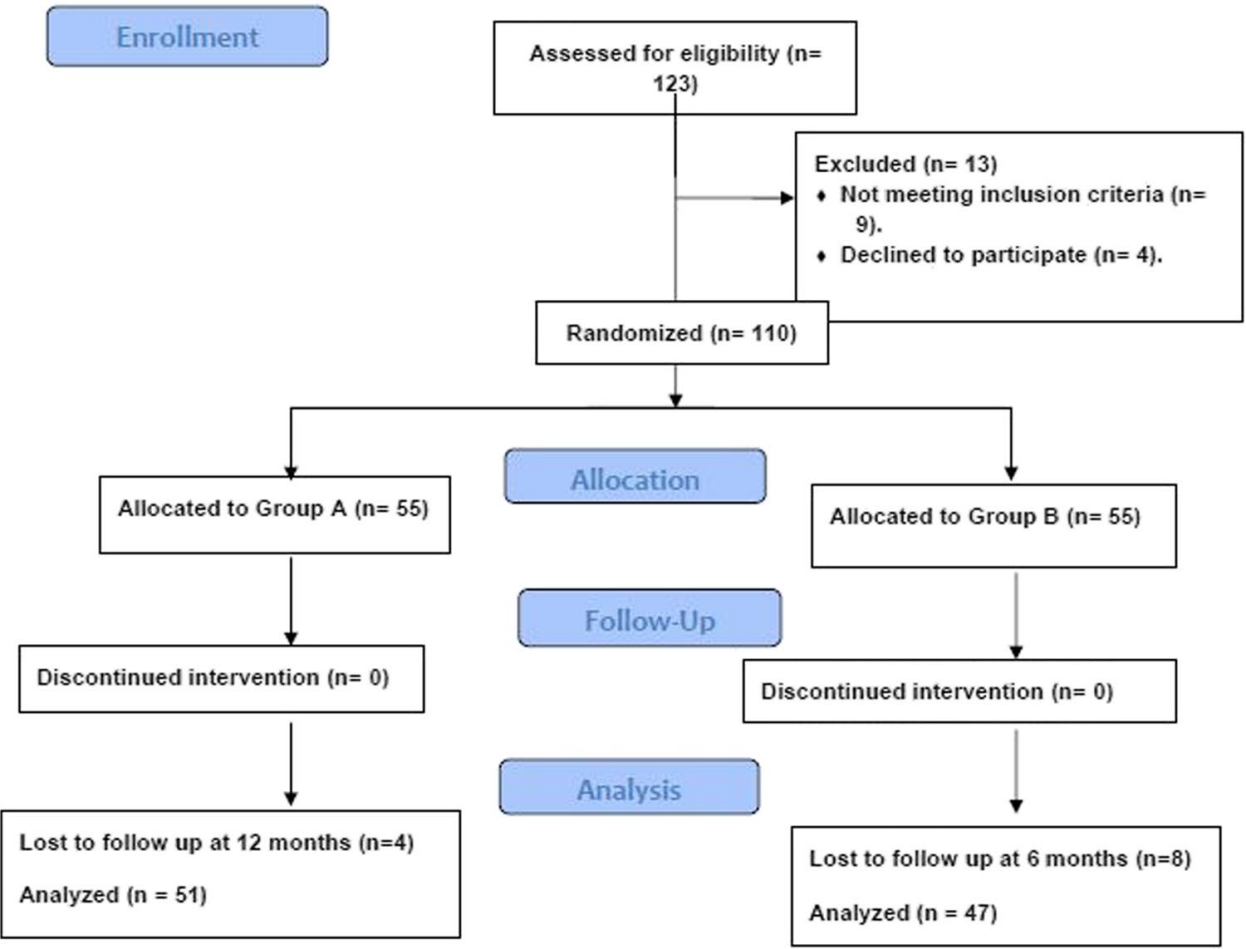
CONSORT flow chart of the study patients

This RCT included 110 patients who were eligible for the study and were equally randomized to the LRYGB-LBPL and LRYGB-SBPL groups. Four patients of the LRYGB-LBPL group and eight patients of the LRYGB-SBPL group were excluded due to dropouts during follow-up, and finally, the two groups included 51 and 47 patients, respectively (Fig. 1).

The mean patients’ age in the LRYGB-LBPL group was 38.42 ± 8.75 years and in the LRYGB-SBPL group was 37.31 ± 7.92 years (*p* = 0.511). Patients were predominantly females in both groups (64.71% and 65.96%, respectively, *p* = 0.896). The mean interval from the primary procedure was 6.94 ± 3.88 years and 7.12 ± 3.47 years (*p* = 0.809), the mean preoperative primary surgery-related EWL% was 33.78 ± 10.91 and 34.83 ± 13.12 (*p* = 0.669), and the mean BMI prior to the conversional surgery was 40.16 ± 7.54 and 41.97 ± 7.33 (*p* = 0.231) in the two groups, respectively.

Dyslipidemia was the most predominant obesity-associated medical complication (33.33% in the LRYGB-LBPL and 23.40% in the LRYGB-SBPL group, *p* = 0.277), followed by hypertension (29.41% in the LRYGB-LBPL and 19.15% in the LRYGB-SBPL group, *p* = 0.238), and diabetes mellitus (23.53% in the LRYGB-LBPL and 14.89% in the LRYGB-SBPL group, *p* = 0.280).

The causes of surgical conversion in the study patients were weight regain (25.49% and 21.28%, *p* = 0.623), inadequate weight loss (70.59% and 72.34%, *p* = 0.848), and intractable GERD (13.73% and 10.64%,* p* = 0.641).

The mean baseline mean arterial pressure (MAP) was 91.77 ± 11.36 and 92.93 ± 13.51 (*p* = 0.648), the mean glycated hemoglobin (HbA1c) levels were 6.45 ± 1.07 and 6.15 ± 0.85 (*p* = 0.306), the mean total cholesterol (TC) levels were 201.8 ± 51.9 and 216.02 ± 49.36 (*p* = 0.168), the mean low-density lipoprotein (LDL) levels were 118.34 ± 18.77 and 120.36 ± 19.02 (*p* = 0.598), and the mean high-density lipoprotein (HDL) levels were 44.86 ± 11.22 and 47.11 ± 12.89 (*p* = 0.361).

The two groups showed no statistically significant differences in the baseline sociodemographic or clinical data (Table 1).

**Table 1:** Baseline demographic data of the study patients (prior to the revisional surgery)

		The LRYGB-LBPL(***n* = **51)	The LRYGB-SBPL** (*n* = **47)	***p*-**value
		Mean ± SD	Mean ± SD	
Age (year)		38.42 ± 8.75	37.31 ± 7.92	0.511^a^
Interval between primary and revision surgery (years)		6.94 ± 3.88	7.12 ± 3.47	0.809^a^
Preoperative BMI (Kg/m^2^)		40.16 ± 7.54	41.97 ± 7.33	0.231^a^
Preoperative EWL%		33.78 ± 10.91	34.83 ± 13.12	0.669^a^
Preoperative MAP (mmHg)		91.77 ± 11.36	92.93 ± 13.51	0.648^a^
Preoperative HbA1c (%)		6.35 ± 1.07	6.15 ± 0.85	0.306^a^
Preoperative TC (mg/dl)		201.8 ± 51.9	216.02 ± 49.36	0.168^a^
Preoperative LDL (mg/dl)		118.34 ± 18.77	120.36 ± 19.02	0.598^a^
Preoperative HDL (mg/dl)		44.86 ± 11.22	47.11 ± 12.89	0.361^a^
		Count (%)	Count (%)	
Sex	Male	18 (35.29%)	16 (34.04%)	0.897^b^
	Female	33 (64.71%)	31 (65.96%)	
Associated medical conditions				
Type 2 diabetes mellitus		12 (23.53%)	7 (14.89%)	0.280^c^
Hypertension		15 (29.41%)	9 (19.15%)	0.238^c^
Dyslipidemia		17 (33.33%)	11 (23.40%)	0.277^c^
Bronchial asthma		1 (1.96%)	2 (4.26%)	0.510^c^
Hypothyroidism		0 (0%)	1 (2.13%)	0.470^c^
Cause of revision				
Weight regains		13 (25.49%)	10 (21.28%)	0.623^c^
In-adequate weight loss		36 (70.59%)	34 (72.34%)	0.848^c^
Intractable GERD		7 (13.73%)	5 (10.64%)	0.641^c^

*BMI*, body mass index; *EWL*, excess weight loss; *MAP*, mean arterial blood pressure; *HbA1c*, hemoglobin A1c; *TC*, total cholesterol; *LDL*, low-density lipoprotein; *HDL*, high-density lipoprotein

^a^Independent t-test

^b^Chi-square test

^c^z test for proportion

### Perioperative Data (Table 2)

**Table 2:** Operative and postoperative data of the study patients

	The LRYGB-LBPL (***n* = **51)	The LRYGB-SBPL (***n* =** 47)	***p***-value
	Mean ± SD	Mean ± SD	
Operative time (minutes)	123.75 ± 9.11	125.54 ± 8.45	0.317^a^
LOS (days)	2.2 ± 0.5	2.1 ± 0.4	0.279^a^
1-year EWL%	67.74 ± 12.32	64.11 ± 10.55	0.122^a^
1-year BMI (Kg/m^2^)	31.6 ± 4.99	33.42 ± 4.76	0.068^a^
1-year EBMIL%	56.46 ± 6.14	50.38 ± 5.17	< 0.001*^a^
1-year MAP (mmHg)	81.43 ± 11.63	81.66 ± 11.03	0.920^a^
Percentage of MAP reduction	11.27% ± 3.90	12.13% ± 3.84	0.275^a^
1-year HbA1c (%)	5.27 ± 0.52	5.42 ± 0.64	0.204^a^
Percentage of HbA1c reduction	17.01% ± 4.53	11.87% ± 4.18	< 0.001*^a^
1-year TC (mg/dl)	173.16 ± 44.8	188.07 ± 40.74	0.089^a^
Percentage of TC reduction	14.19% ± 5.18	12.94% ± 4.07	0.190^a^
1-year LDL (mg/dl)	102.4 ± 21.25	105.75 ± 18.35	0.407^a^
Percentage of LDL reduction	13.47% ± 4.12	12.14% ± 4.47	0.129^a^
1-year HDL (mg/dl)	57.44 ± 5.65	52.46 ± 4.81	< 0.001*^a^
Percentage of HDL increase	28.04% ± 6.88	11.36% ± 5.86	< 0.001*^a^
Complete remission of comorbidities			
Type 2 diabetes mellitus	9/12 (23.53%)	4/7 (14.89%)	0.183^b^
Hypertension	10/15 (29.41%)	5/9 (19.15%)	0.218^b^
Dyslipidemia	15/17 (33.33%)	8/11 (23.40%)	0.148^b^

*BMI*, body mass index; E*WL*, excess weight loss; *MAP*, mean arterial blood pressure; *HbA1c*, hemoglobin A1c; *TC*, total cholesterol; *LDL*, low-density lipoprotein; *HDL*, high-density lipoprotein

^a^Independent t-test

^b^Chi-square test

^c^z test for proportion

^*^Statistically significant

The mean operative time was comparable in the two groups (123.75 ± 9.11 min in the LRYGB-LBPL group and 125.54 ± 8.45 min in the LRYGB-SBPL group, *p* = 0.317). The mean length of hospital stay was 2.2 ± 0.5 days and 2.1 ± 0.4 days in the two groups, respectively, with no statistically significant difference (*p* = 0.279).

### 12-Month Follow-up Data

**Fig. 2:**
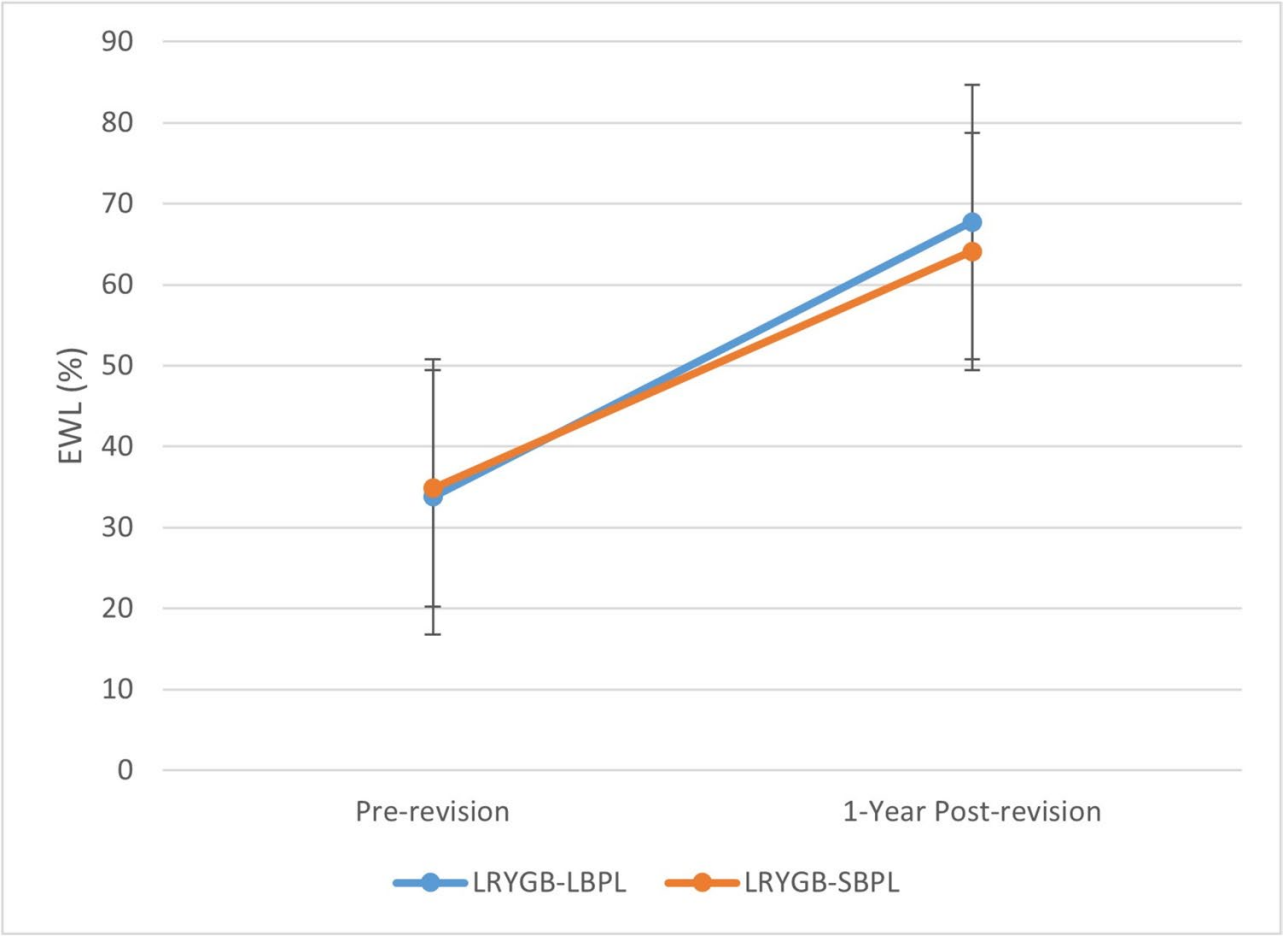
EWL% in the two groups

At the 12-month follow-up, the two study groups showed a statistically significant reduction in BMI, with a 12-month mean BMI of 31.6 ± 4.99 in the LRYGB-LBPL group (*p* < 0.001) and 33.42 ± 4.76 in the LRYGB-SBPL group (*p* < 0.001). No statistically significant differences were noted in the 12-month BMI or EWL% (Fig. 2). The mean EBMIL% was significantly higher in the LRYGB-LBPL group compared to the LRYGB-SBPL group (*p* < 0.001).

The LRYGB-LBPL group revealed a higher complete remission rate of dyslipidemia (33.33% vs. 23.40%, *p* = 0.148), hypertension (29.41% vs. 19.15%, *p* = 0.218), and diabetes mellitus (23.53% vs. 14.89%, *p* = 0.183), with no statistically significant differences.

All patients with GERD in the two groups showed improvement, with mild symptoms remaining in one patient in the LRYGB-LBPL group (1/7, 14.29%) and in two patients in the LRYGB-SBPL group (2/5, 40%). The difference was statistically not significant (*p* = 0.523).

The LRYGB-LBPL group showed a statistically significant higher percentage of HbA1c reduction (17.01% vs. 11.87%, *p* < 0.001), higher mean HDL levels (57.44 ± 5.65 vs. 52.46 ± 4.81, *p* < 0.001), and a higher percentage of HDL increase (28.04% vs. 11.36%, *p* < 0.001). The percentages of improvement in MAP, TC, and LDL were comparable in the two groups (*p* > 0.05).

### Postoperative Complications (Table 3)

**Table 3:** Postoperative complications of the study patients

		The LRYGB-LBPL** (*n* = 51)**	**The LRYGB-SBPL (** ***n*** ** = 47)**	***p*** **-value**
		*n* (%)	*n* (%)	
General	Bleeding	2 (3.92%)	2 (4.26%)	1.000^d^
	Wound infection	2 (3.92%)	3 (6.38%)	0.722^d^
	Chest infection	1 (1.96%)	1 (2.13%)	1.000^d^
	Leakage	1 (1.96%)	0 (0.00%)	0.335^d^
	Port site hernia	1 (1.96%)	0 (0.00%)	0.335^d^
	DVT	3 (5.88%)	2 (4.26%)	0.720^d^
	ICU admission	2 (3.92%)	1 (2.13%)	1.000^d^
Metabolic	Dumping syndrome	5 (9.80%)	4 (8.51%)	1.000^d^
Nutritional	Anemia	2 (3.92%)	3 (6.38%)	0.665^d^
	Hypoalbuminemia	1 (1.96%)	2 (4.26%)	0.660^d^
	Zinc deficiency	1 (1.96%)	1 (2.13%)	1.000^d^
	Selenium deficiency	0 (0.00%)	0 (0.00%)	1.000^d^
	Copper deficiency	0 (0.00%)	0 (0.00%)	1.000^d^
	Vitamin B12 deficiency	1 (1.96%)	1 (2.13%)	1.000^d^
	Vitamin D deficiency	2 (3.92%)	4 (8.51%)	0.538^d^
	Total	4 (7.84%)	2 (4.26%)	0.660^d^
Total patients’ number		11 (21.57%)	8 (17.02%)	0.569^b^

*BMI*, body mass index; *EWL*, excess weight loss; *MAP*, mean arterial blood pressure; *HbA1c*, hemoglobin A1c; *TC*, total cholesterol; *LDL*, low-density lipoprotein; *HDL*, high-density lipoprotein

^a^Independent t-test

^b^Chi-square test

^d^Fisher’s exact test

No statistically significant differences were noted between the two groups in the incidence of postoperative complications (21.57% in the LRYGB-LBPL group and 17.02% in the LRYGB-SBPL group, *p* = 0.569). These complications encompassed a range of general surgical issues, including bleeding (3.92% vs. 4.26%), wound infections (3.92% vs. 6.38%), chest infections (1.96% vs. 2.13%), leakage (1.96% vs. 0.00%), port site hernias (1.96% vs. 0.00%), deep vein thrombosis (DVT) (5.88% vs. 4.26%), and ICU admissions (3.92% vs. 2.13%). Additionally, metabolic and nutritional complications such as dumping syndrome (9.80% vs. 8.51%), anemia (3.92% vs. 6.38%), hypoalbuminemia (1.96% vs. 4.26%), zinc deficiency (1.96% vs. 2.13%), selenium deficiency (0.00% vs. 0.00%), copper deficiency (0.00% vs. 0.00%), vitamin B12 deficiency (1.96% vs. 2.13%), and vitamin D deficiency (3.92% vs. 8.51%) were recorded. No significant differences were found between the two groups for any individual complication (all *p* > 0.05).

## Discussion

In parallel with the steadily rising popularity of SG, which is attributed to its technical ease, efficacy, and safety, there have been growingly increasing rates of conversional surgeries due to inadequate weight loss, weight re-gain, or surgery-related complications [6–9]. Although RYGB has been the gold standard for conversion of primary restrictive MBSs with SoCR or RWG [8, 9], no studies, to our knowledge, have investigated the potential impact of the BPL length on the outcome of conversional RYGB in a head-to-head comparative RCT.

The different absorptive capacities of BPL and AL have been described with heterogeneous results and continuous debates regarding the best choices [20, 21]. While AL likely retains some absorptive capacity, BPL may experience a significant reduction in nutrient absorption. Furthermore, different, not fully understood hormonal mechanisms have been related to the variation in the outcomes of these limbs’ lengths [12]. Therefore, it is important to determine the optimal lengths of the BPL and AL that can be safely bypassed without causing significant nutritional deficiencies. In the present study, we used a BPL length of 150 cm with an AL length of 75 cm versus a BPL of 50–75 cm with an AL length of 150 cm.

The comparative analysis between the two groups revealed non-significant differences in the mean operative time or LOS, indicating that the BPL length did not significantly impact the duration of the surgical procedure or hospitalization duration. This is similar to the prospective randomized trial by Zerrweck et al. [12], who found no significant difference in the duration of surgery or the stay in hospital between short- and long-limb RYGB in patients.

This study also evaluated early postoperative complications as well as late nutritional complications, with the LRYGB-LBPL group experiencing an overall complication rate slightly higher than that found in the LRYGB-SBPL group with no significant difference. Several studies have compared the postoperative complications associated with short- and long-limb RYGB. In consistency with our results, Zerrweck et al. [12], Inabnet et al. [22], and Dogan et al. [23] found no significant difference in overall short-term and long-term complication rates.

In the present study, at the 12-month follow-up, both the LRYGB-LBPL and LRYGB-SBPL groups demonstrated a substantial weight loss outcome in terms of reduction in BMI and achieving optimum percentages of EWL. However, a notable distinction between the two groups was observed in the EBMIL%, where the LRYGB-LBPL group exhibited a significantly higher mean EBMIL% compared to the LRYGB-SBPL group.

Research comparing the weight loss outcomes between long and short BPL in RYGB has produced mixed results. According to Eckharter et al. [24], no statistically significant difference in the reduction of weight was observed between long and short BPLs in RYGB, with an EWL% and TWL% being comparable. Similarly, Kamocka et al. [25] found no conclusive evidence that altering the BPL length significantly affects weight loss outcomes. In contrast, Nijland et al. [26] reported that patients with a long BPL had a significantly higher TWL% than those with a short BPL up to 4 years after surgery. Shah et al. [27] also noted that the distalization of RYGB with longer BPL resulted in improved weight loss outcomes. Other studies by Nergaard et al. [28] and Nora et al. [29] found that a longer BPL was related to a significantly higher EBMIL%.

The inconsistencies across studies may be due to variations in study design, patient populations, follow-up durations, and the parameters used to assess the degree of weight loss. Differences in baseline characteristics, such as initial BMI, comorbid conditions, and adherence to postoperative lifestyle modifications, can also contribute to the varying outcomes observed. Additionally, the degree of malabsorption induced by different BPL lengths may vary between individuals, influencing the overall effectiveness of the procedure.

The metabolic effects of varying BPL lengths in RYGB have been a matter of extensive discussion. Interestingly, the present study showed that, despite the LRYGB-LBPL-associated higher complete remission rates of dyslipidemia, hypertension, and diabetes mellitus not reaching the significance levels, a notable and statistically significant higher reduction in HbA1c levels, indicating a more substantial improvement in glycemic control, higher mean HDL levels, and a greater percentage of elevation in HDL were shown in the LRYGB-LBPL group, signifying a favorable impact on cardiovascular health.

In this context, Ke et al. [30] observed that extending the BPL length to 100 cm had no significant effect on glycemic control compared to shorter lengths, with similar improvements in glucose and lipid metabolic parameters across both groups, whereas Kaska et al. [31] found that a BPL length of 100–150 cm was associated with a higher rate of diabetes remission. On the same line, Homan et al. [32], in an RCT, found that RYGB with a BPL length of 150 cm and an AL length of 75 cm showed a higher rate of dyslipidemia remission than surgery with a BPL length of 75 cm and an AL length of 150 cm. Nora et al. [29] and Zerrweck et al. [12] also concluded better diabetes remission in patients who underwent long BPL RYGB. Eskandaros and Abbass [33] concluded the superiority of long BPL RYGB in both diabetes and dyslipidemia remission.

Overall, these study results emphasize that LRYGB-LBPL was relatively advantageous with better weight loss and metabolic improvement in certain aspects, reflecting the more enhanced malabsorptive capacity associated with the longer BP without significantly higher risks of complications. This aligns with the trending emphasis on the importance of BPL length for its metabolic benefits [29]. Elongation of the BPL has been assumed to exceed simply accentuating malabsorption. A long BPL-associated hormonal profile is characterized by a reduction in insulin levels that coincides with an increase in glucagon-like peptide-1 levels during fasting and postprandial [11]. This profile has been proposed to be mediated by the effect of food entry to the ileum with an alteration in alimentary behavior and the differential impact of a longer BPL on immunological and hormonal factors [11, 31, 32, 34].

This study’s limitations include a short-term 12-month follow-up, potentially limiting the understanding of long-term effects, and being a single-center study. One of the limitations of our study is the reliance on a structured symptom-based scoring system without routine objective testing such as esophagogastroduodenoscopy (EGD). While our approach provides valuable insights into symptom severity, it may not capture asymptomatic esophageal mucosal alterations. Further research exploring the long-term outcomes and quality of life improvements associated with each technique as well as incorporating routine objective assessments to provide a more comprehensive evaluation of GERD will be unique in refining metabolic and bariatric surgical practices and optimizing patient care.

Additionally, the study was not blinded, which may have introduced a degree of observer bias in outcome assessments. Furthermore, the absence of a formal quality-of-life measure limits the assessment of patient-reported outcomes.

While our study utilized the prevailing definitions for specific cause of revision (SoCR and RWG) at the time of data collection, it is acknowledged that newer definitions proposed by the latest IFSO consensus [35] may offer a more nuanced understanding of the indications for revisional RYGB. Future research should aim to incorporate these updated criteria prospectively to refine patient selection and optimize surgical outcomes. Nevertheless, our findings provide valuable insights into the efficacy of limb length variations in managing inadequate weight loss and GERD, which remain pertinent irrespective of evolving definitional frameworks.

In addition, we acknowledge that the relatively small sample size may have limited our ability to detect a significant difference in the nutritional complications which showed a trend toward a higher incidence in patients undergoing the long BP RYGB procedure compared to the short BP approach. We suggest that future investigations with larger, multicenter cohorts are warranted to confirm these findings and further explore the clinical implications. The observed clinical trend necessitates careful consideration. We recommend a detailed nutritional evaluation and patient counseling to identify and address potential deficiencies before surgery and to develop individualized supplementation regimens based on periodic nutritional assessments and current clinical guidelines.

## Conclusion

In conclusion, this study highlights the potential benefits of using a longer BPL length in conversional RYGB surgery. While operative time, hospital stay, and overall complication rates were similar between groups, the long BPL group demonstrated statistically significant improvements in EBMIL%, HbA1c reduction, and HDL increase. These findings suggest that limb length modification may offer metabolic advantages in selected patients without added risk of complications.

## Data Availability

No datasets were generated or analysed during the current study.
